# Canine parvovirus detected from a serval (*Leptailurus serval*) in South Africa

**DOI:** 10.4102/jsava.v90i0.1671

**Published:** 2019-03-25

**Authors:** Almero Oosthuizen, Helene Brettschneider, Desire L. Dalton, Elizabeth C. du Plessis, Raymond Jansen, Antoinette Kotze, Emily P. Mitchell

**Affiliations:** 1 Department of Research and Specialised Services, National Zoological Gardens, South African National Biodiversity Institute, Pretoria, South Africa; 2 Department of Environmental, Water and Earth Sciences, Faculty of Science, Tshwane University of Technology, Pretoria, South Africa; 3 School of Mathematical and Natural Sciences, University of Venda, Thohoyandou, South Africa; 4 IDEXX Laboratories (Pty) Ltd, Faculty of Veterinary Science, University of Pretoria, Pretoria, South Africa; 5 Department of Genetics, University of the Free State, Bloemfontein, South Africa; 6 Department of Pathology, Faculty of Veterinary Science, University of Pretoria, Pretoria, South Africa

## Abstract

Canine parvovirus first emerged in domestic dogs (*Canis familiaris*), most likely as a variant of the feline panleucopaenia virus. Relatively recently, canine parvovirus-2a and canine parvovirus-2b infections have been identified in both symptomatic and asymptomatic domestic cats, while canine parvovirus infections have also been demonstrated in wild felids. This report documents the first known case of canine parvovirus-2b detected in unvaccinated serval (*Leptailurus serval*) from South Africa. The serval presented with clinical signs of vomiting, anorexia and diarrhoea that responded to symptomatic treatment. Two weeks later, severe leucopaenia, thrombocytopenia and death occurred. Typical enteric histological lesions of parvovirus infection were not observed on histopathological examination of the small intestine; however, histological lesions consistent with septicaemia were present. Canine parvovirus was detected in formalin-fixed paraffin-embedded small intestine using polymerase chain reaction. Phylogenetic analysis of the sequence of the canine parvovirus viral capsid protein gene showed similarities between the sample from the serval and canine parvovirus-2b isolates from domestic dogs in Argentina and South Africa. A case of canine parvovirus-2b in a domestic dog from South Africa in 2012 that fell within the same clade as the serval sample appears distantly related because of the long branch length. The significance of these findings is explored. More extensive surveys of canine parvovirus in domestic and wild felids and canids are needed to understand the epidemiology of canine parvovirus in non-domestic felids in South Africa.

## Introduction

Parvoviruses (family *Parvoviridae*) and their associated diseases affect various carnivores including felids and canids (Siegl et al. 1985). Feline panleucopaenia virus (FPLV) infection was one of the first viral diseases identified in domestic cats (*Felis catus*), during the 1930s and 1940s, and also infects non-domestic felids (Parrish 1990). At roughly the same time (~1947), parvoviral gastroenteritis was identified in farmed mink (*Mustela vison*), and within 15 years, it was observed on mink ranches throughout Canada, the United States, Europe and Scandinavia (Parrish 1990; Pearson & Gorham 1987); it is currently known as mink enteritis virus (MEV) (Barker & Parrish 2001).

During the late 1970s, canine parvovirus (CPV) first emerged in domestic dogs (*Canis familiaris*), most likely as a variant of FPLV or a closely related parvovirus. Although the exact mechanism of emergence is unclear, amplification of parvovirus DNA sequences intermediate between FPLV and CPV from red foxes (*Vulpes vulpes*) suggests that wildlife may have played a role in the adaptation of the virus in a new host (Parrish 1995; Truyen et al. 1996, 1998). The original strain (CPV type 2) spread worldwide rapidly, shortly thereafter being replaced by type 2a and a few years later by type 2b. These two variants, however, differ very little from the original strain (Parrish 1995; Truyen et al. 1996). Strain CPV type 2c was diagnosed much later (~2000), from domestic dogs in Italy, and has been proven to induce disease in cats as well (Buonavoglia et al. 2001; Nakamura et al. 2001).

Host range seems to be complex, as even within affected families only certain genera or species have been reported to be susceptible (Barker & Parrish 2001). The natural hosts of CPV (dogs), FPLV (cats) and MEV (mink) have been identified; however, the natural host ranges of feline subgroup parvoviruses are poorly defined (Barker & Parrish 2001). Wild felids that have been reported to be susceptible to FPLV include cheetah (*Acinonyx jubatus*), Siberian tiger (*Panthera tigris altaica*), African wildcat (*Felis lybica*), puma (*Puma concolor*), ocelot (*Leopardus pardalis*) and spotted cat (*Leopardus tigrinus*) (Filoni et al. 2006; Hoelzer & Parrish 2010; Steinel et al. 2000). In addition, FPLV has been reported in many non-felid species, for example raccoon (*Procyon lotor*), mink (*Neovison vison*) and arctic fox (*Vulpes lagopus*) (Hoelzer & Parrish 2010; Steinel et al. 2001; Van Vuuren et al. 2000).

Domestic cats have been shown to be both symptomatic and asymptomatic carriers of CPV-2a, 2b and 2c (Buonavoglia et al. 2001; Clegg et al. 2012; Nakamura et al. 2001), while infection has also been demonstrated in wild felids such as cheetah and Siberian tiger (Steinel et al. 2000). Similarly, both domestic dogs and wild canids are known to be susceptible to CPV, while antibodies against CPV have been found in coyote (*Canis latrans*), grey wolf (*Canis lupus*), maned wolf (*Chrysocyon brachyurus*), crab-eating fox (*Cerdocyon thous*), bush dog (*Speothos venaticus*), dingo (*Canis lupus dingo*), raccoon dog (*Nyctereutes procyonoides*) and African wild dog (*Lycaon pictus*) (Alexander et al. 1993; Barker & Parrish 2001; Van Heerden et al. 1995).

Genetic material from FPLV has been detected in a serval (*Leptailurus serval*) with histological lesions typical for parvoviral disease and in another with salmonellosis (Lane et al. 2016). This case explores the significance of the detection of CPV-2b in a serval from South Africa with bacterial septicaemia.

## Materials and methods

### Case presentation

A captive adult male serval that presented with inappetence, anorexia, vomiting and diarrhoea was hospitalised at a veterinary practice in Nelspruit, Mpumalanga. The animal reacted well to symptomatic treatment and regained its appetite. Fifteen days later, however, it presented with epistaxis, mild icterus, anaemia (haematocrit = 28), severe leucopaenia and thrombocytopenia (subjectively evident on a blood smear). Treatment was initiated but the animal died the following day. No evidence of warfarin or other anticoagulant exposure was reported. No further clinical tests or culture were performed, and no bone marrow was submitted for evaluation or further analysis.

A necropsy showed that the serval was in good condition with mild icterus, a slightly enlarged liver and mild fluid accumulation in the body cavities. The cause of death was suspected to be because of aspiration of blood from the epistaxis. Selected tissue samples preserved in 10% buffered formalin were processed routinely and stained with haematoxylin–eosin stain. The histopathological examination confirmed septicaemia (multifocal hepatic and pulmonary necrosis associated with fine bacterial bacilli, and fibrin deposition in the splenic red pulp sinuses). Hepatic macrophages contained bile pigment in the cytoplasm. The urinary bladder mucosa showed multifocal mucosal ulceration and haemorrhage with mild submucosal lymphoplasmacytic interstitial infiltration and fibroplasia. An unspecified lymph node was mildly active and cortical follicular structures contained central hyalinisation of the stroma and possible fibrin deposition. The small intestine did not reveal any enteric lesions including changes characteristic of parvovirus-induced necrosis.

The molecular diagnostics and phylogenetic analyses formed part of a larger project at the National Zoological Garden, South African National Biodiversity Institute (NZG, SANBI), which investigates the molecular identification and genetic diversity of FPLV in both wild and domestic felids (Lane et al. 2016). Here, we compared the serval sample (PV19) to other cases, which include PV0 to PV30 (various felids, Lane et al. 2016), PV31 (a domestic dog from Namibia) and PV35, PV36, PV38 and PV40 (domestic dogs from Pretoria) as summarised in Table 1. No domestic dog samples from Mpumalanga were available to include in the report.

**TABLE 1:** Sample type, species, histopathological diagnosis and molecular analysis of parvoviral samples collected as part of the National Zoological Garden, South African National Biodiversity Institute wildlife disease database.

Lab no.	Sample type	Species	Histopathological diagnosis†	Molecular analysis
PV10‡	F/F	Serval	Suspected non-FPLV	FPLV Clade 1
PV15‡	FFPE	Serval	Suspected FPLV	FPLV Clade 2
PV19	FFPE	Serval	Suspected FPLV	CPV-2b
PV36	FFPE	Domestic dog	CPV	CPV-2a
PV38	FFPE	Domestic dog	CPV	CPV-2a
PV40	FFPE	Domestic dog	CPV	CPV-2a
PV31	FFPE	Domestic dog	CPV	CPV-2b
PV35	FFPE	Domestic dog	CPV	CPV-2b
PV1‡	F/F	African black footed cat	FPLV	FPLV Clade 1
PV24‡	FFPE	Caracal	Suspected FPLV	FPLV Clade 1
PV0‡	F/F	Cheetah	Suspected FPLV	FPLV Clade 1
PV4‡	FFPE	Cheetah	FPLV	FPLV Clade 1
PV7‡	FFPE	Cheetah	FPLV	FPLV Clade 1
PV8‡	FFPE	Cheetah	FPLV	FPLV Clade 1
PV9‡	FFPE	Cheetah	FPLV	FPLV Clade 1
PV11‡	F/F	Cheetah	FPLV	FPLV Clade 1
PV12‡	F/F	Cheetah	FPLV	FPLV Clade 1
PV26‡	F/F	Cheetah	FPLV	FPLV Clade 1
PV27‡	Rectal swab	Cheetah	FPLV	FPLV Clade 1
PV28‡	F/F	Cheetah	FPLV	FPLV Clade 1
PV29‡	F/F	Cheetah	FPLV	FPLV Clade 1
PV14‡	FFPE	Lion	FPLV	FPLV Clade 1
PV23‡	FFPE	Lion	FPLV	FPLV Clade 1
PV21‡	FFPE	Ocelot	Suspected FPLV	FPLV Clade 1
PV30‡	FFPE	Caracal	Suspected FPLV	PCR negative
PV3‡	F/F	Cheetah	Suspected FPLV	PCR negative
PV5‡	F/F	Cheetah	FPLV	PCR negative
PV13‡	FFPE	Cheetah	Suspected FPLV	PCR negative
PV2‡	F/F	Domestic cat	Suspected FPLV	PCR negative
PV20‡	F/F	Leopard	Non-FPLV	PCR negative
PV6‡	F/F	Lion	Suspected FPLV	PCR negative
PV17‡	F/F	Lion	Suspected FPLV	PCR negative
PV22‡	FFPE	Lion	FPLV	PCR negative
PV25‡	F/F	Ocelot	Non-FPLV	PCR negative
PV18‡	FFPE	Puma	Non-FPLV	PCR negative

FFPE, formalin-fixed paraffin-embedded; F/F, fresh or frozen; FPLV, feline panleucopaenia virus; CPV, canine parvovirus; PCR, polymerase chain reaction.

† , Histopathological diagnosis was made on the presence of diagnostic or strongly suggestive lesions for parvoviral infection.

‡ , Samples from Lane et al. (2016).

Nucleic acids from a formalin-fixed, paraffin-embedded (FFPE) intestinal sample (PV19) were extracted using the Epicentre MasterPure™ Complete DNA & RNA Purification Kit (Whitehead Scientific^®^) following the manufacturer’s specifications for FFPE tissue, and deparaffinisation of FFPE samples was performed using xylene.

Molecular analysis of parvovirus from the isolate was achieved by amplification of a ~1400 bp region of the viral capsid protein (VP2) gene (Table 2) using DreamTaq™ Green PCR (polymerase chain reaction) master mix (ThermoFischer Scientific) and previously published primers (Horiuchi et al. 1996; Meers et al. 2007; Steinel et al. 2000; Wasieri et al. 2009). A standard polymerase chain reaction (PCR) setup was used consisting of 8.5 *µ*L nuclease-free water (supplied with DreamTaq™), 12.5 *µ*L DreamTaq master mix, 1 *µ*L of each primer (Forward and Reverse; 10 pmol) and 2 *µ*L DNA sample standardised to a concentration of 50 ng/*µ*L. Cycling conditions for tissue isolates using primer sets 2 to 5 were as follows: initial denaturation (94 °C for 5 minutes), 30 cycles of denaturation (94 °C for 30 seconds), annealing (58 °C for 50 s) and extension (72 °C for 1 min). A final extension step (72 °C for 10 min) concluded the cycling. Cycling conditions for primer sets 1 and 6 were identical except annealing occurred at 54 °C. Conditions were adjusted for FFPE isolates by decreasing the annealing temperatures to 54 °C for primer sets 2 to 5, and 50 °C for primer sets 1 and 6. Polymerase chain reaction setup was performed in a DNA-free hood and a DNA negative control was included.

**TABLE 2:** Details of sequences and amplicon size of primers used in this study for polymerase chain reaction and sequencing spanning the viral capsid protein 2 gene region.

Primer set	Name	Sequence (5’–3’)	Amplicon size (base pair)
1	CPV_EF†	GCCGGTGCAGGACAAGTA	421
	M2R‡	AGTTGCCAATCTCCTGGATT	
2	PV_VP2-1§	GAAAACGGATGGGTGGAAATC	405
	PV_VP2-2§	AGCTGCTGGAGTAAATGGCATAGT	
3	PV_VP2-3§	TAAAGACTGTTTCAGAATCTGCTACTCA	424
	PV_VP2-4§	AGAAATGGTGGTAAGCCCAATG	
4	PV_VP2-5§	ATACTGGAACTAGTGGCACACC	437
	PV_VP2-5ii¶	ATTTAAAACACCTATTGCAGCAGGAC	
5	PV_VP2-5iiR¶	GTCCTGCTGCAATAGGTGTTTTAAAT	440
	PV_VP2-8§	GCATCAGGATCATATTCATTTGTTAA	
6	Primer 22 F††	TGTCAAAATAATTGTCCTGG	564
	CPV_JS2R†	CAACCCACACCATAACAACA	

PV, Parvovirus; VP2, viral capsid protein 2.

† , Meers et al. 2007.

‡ , Steinel et al. 2000.

§ , Wasieri et al. 2009.

¶ , Helene Brettschneider.

†† , Horiuchi et al. 1996.

All positive amplicons were purified using an exonuclease I and alkaline phosphatase PCR purification protocol, sequenced using BigDye V3.1 (Applied Biosystems) chemistry and analysed on an ABI3500 genetic analyser. Sequences were aligned using the ClustalX function incorporated in MEGA6 (Tamura et al. 2011) and phylogenetically analysed using neighbour-joining (NJ) in MEGA6. The Tamura 3-parameter model with gamma distribution (T92+G; G = 0.12) was determined to be the best-fit model of sequence evolution under the Akaike Information Criterion (AIC) in jModeltest (Posada 2008). Phylogenetic analyses were performed on 61-taxon, trimmed to the shortest sequence (~1200 bp), VP2 gene data set including 11 FPLV positive isolates, two FPLV vaccine strains (Fel-o-vax IV^®^, Boehringer Ingelheim; Felocell^®^, Zoetis), six CPV positive case samples and 42 parvovirus reference strains (Table 3) from the National Centre for Biotechnology Information (NCBI) online database, GenBank (https://www.ncbi.nlm.nih.gov/). Identical field isolate sequences were selectively removed, to reduce the phylogenetic tree size, so that only representative isolates remained.

**TABLE 3:** GenBank reference sequences including species, country of origin, year of collection and accession number.

Species	Virus	Collection origin and year	Accession number
Cell line	CPV	Unknown; 1990	M38245
Domestic dog	CPV-2	ITA; 2005	FJ222824
Vaccine	CPV-2	CHN; 2008	FJ432718
Fox	CPV-2	CHN; 2009	GU392236
Domestic dog	CPV-2	RSA; 2010	HQ602985
Domestic dog	CPV-2	RSA; 2010	HQ602986
Cell line	CPV-2a	CHN; 2007	EU310373
Domestic dog	CPV-2a	CHN; 2011	JQ268283
Domestic dog	CPV-2a	CHN; 2011	JX660690
Domestic dog	CPV-2a	URY; 2011	KC196111
Domestic dog	CPV-2a	CHN; 2009	KF482472
Domestic dog	CPV-2a	CHN; 2011	KF785797
Domestic dog	CPV-2a	CHN; 2014	KT382542
Domestic dog	CPV-2b	ARG; 2003	JF414817
Domestic dog	CPV-2c	DEU; 1997	FJ005196
Domestic dog	CPV-2c	BEL; 2008	FJ005247
Domestic dog	CPV-2c	GRC; 2008	GQ865518
Domestic dog	CPV-2c	URY; 2008	KC196105
Domestic dog	CPV-2c	ITA; 2010	KF373598
Domestic dog	CPV-2c	ITA; 2009	KF385386
Blue fox	BFPV	CHN; 2008	GQ857595
Domestic cat	FPLV	RSA; 1999	AJ249556
Cheetah	FPLV	RSA; 1999	AJ249557
Cell line	FPLV	RUS; 2004	AY665655
Vaccine Purevax	FPLV	Merial; 2008	EU498680
Vaccine Felocell	FPLV	Pfizer; 2008	EU498681
Domestic cat	FPLV	KOR; 2008	HQ184200
Raccoon	FPLV	US; 1978	JN867596
Domestic cat	FPLV	TWN; 2011	JX048608
Cougar	FPLV	US; 2010	JX475253
Domestic cat	FPLV	CHN; 2014	KP280068
Cell line	FPLV	US; 1988	M24002
Cell line	FPLV	Unknown; 1988	M24004
Cell line	FPLV	Unknown; 1990	M38246
Wild cat	FPLV	US; 1990	U22187
Cell line	MEV	RUS; 1998	AF201477
Cell line	MEV	CHN; 2007	EF428258
Mink	MEV	CHN; 2009	GU272028
Mink	MEV	CHN; 2010	JX535284
Mink	MEV	CHN; 2012	KC713592
Mink	MEV	CHN; 2011	KP008112
Mink	MEV	CHN; 2014	KT899745

ARG, Argentina; US, United States; PRT, Portugal; URY, Uruguay; ITA, Italy; BEL, Belgium; CHN, China; GRC, Greece; DEU, Germany; TWN, Taiwan; RSA, South Africa; KOR, Korea; RUS, Russia; CPV, canine parvovirus; FPLV, feline panleucopaenia virus; BFPV, blue fox parvovirus.

### Ethical considerations

Ethical approval was obtained from the NZG Research Ethics and Scientific Committee (NZG/RES/P/16/10).

## Results

**FIGURE 1:**
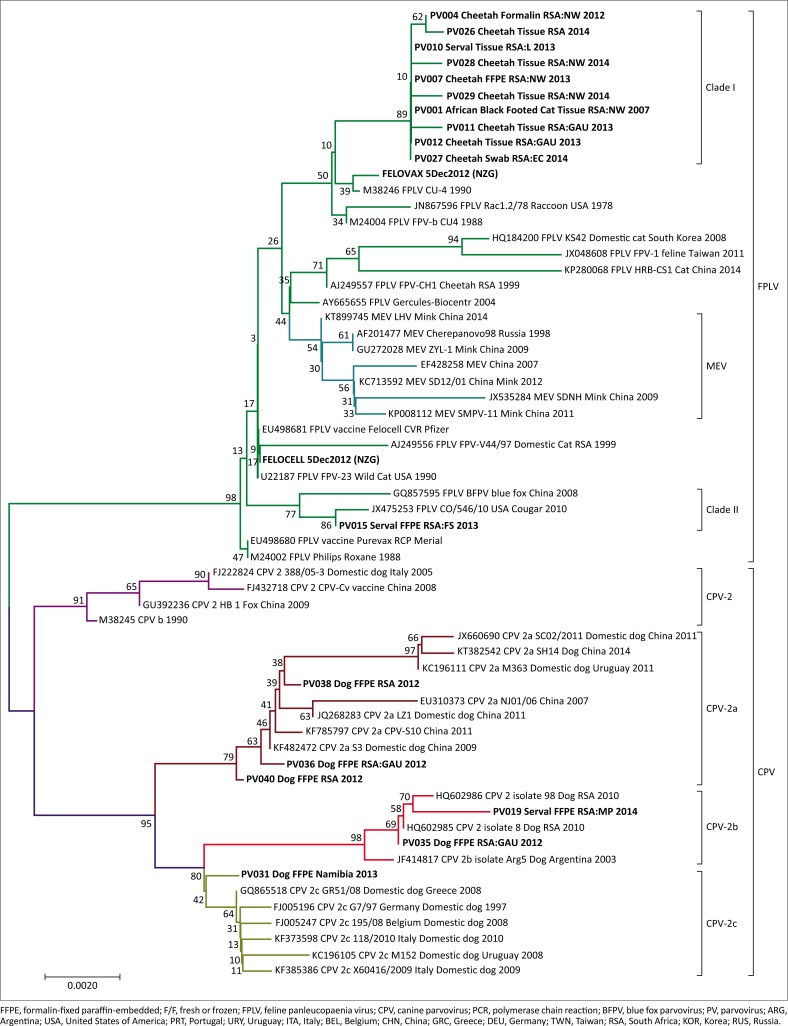
Evolutionary relationships of detected parvoviral taxa using trimmed nucleotide sequences and the neighbour-joining method. The tree was generated using the Tamura 3-parameter model with Gamma distribution and 1000 bootstrap replicates. Cases from the NZG wildlife disease database and the study by Lane et al. (2016) are prefixed with laboratory numbers (PV), vaccine strains are prefixed with the vaccine name, and reference strains collected from GenBank are prefixed with the accession number. Rooted with CPV as the out-group.

Results from the NJ analysis (1000 bootstrap replicates) illustrate a clear separation between FPLV and CPV isolates, as indicated in Figure 1. Additionally, the CPV clade consists of four distinct clusters representing CPV-2, -2a, -2b and -2c. The serval isolate from this study (PV19) grouped with the CPV-2b cluster with a domestic dog sample from the NZG, SANBI database (PV35), and with previously reported NCBI reference sequences from domestic dogs in Argentina (Gallo Calderón et al. 2012) and South Africa (Dogonyaro et al. 2013). Isolate PV19, however, appears to be distantly related to these isolates as indicated by the long branch length. The Namibian domestic dog isolate (PV31) from our database grouped with the CPV-2c cluster on a separate branch. Domestic dog isolates PV36, PV38 and PV40 grouped with the CPV-2a cluster also on separate branches. The majority of the South African felid samples grouped with the FPLV Clade I, including a sample from a serval with salmonellosis (PV10) (Lane et al. 2016). The South African Clade II contained one serval with histological lesions of FPLV (PV15) with a seemingly unique viral strain (Lane et al. 2016) that is more similar to a Felocell vaccine strain than to Clade I FPLV.

## Discussion

This study reports the first detection of CPV-2b in a captive serval from South Africa, while the study by Lane et al. (2016) previously detected FPLV in two serval isolates. Although CPV infection has previously been detected in wild felids, such as cheetah and Siberian tiger (Steinel et al. 2000), as well as domestic cats, the CPV-2b strain detected in the serval corresponds more closely with those detected in domestic dogs from South Africa (Dogonyaro et al. 2013) and Argentina (Gallo Calderón et al. 2012).

The significance of the presence of CPV and FPLV in tissues from servals with septicaemia is uncertain. Parvovirus is known to cause lymphoid necrosis and atrophy in lymphoid organs such as the thymus, gut-associated lymphoid tissue and lymph nodes as well as intestinal epithelial necrosis. The resultant immunosuppression and breach of intestinal integrity make affected animals susceptible to secondary systemic bacterial infection. Typically, domestic dogs and cats that die as a result of septicaemia secondary to parvoviral infection show histological lesions of intestinal epithelial necrosis. Detection of FPLV in a serval (PV15) with typical intestinal necrosis suggests that the disease, in FPLV at least, may show a similar course in servals. However, as domestic cats have been shown to be asymptomatic carriers of CPV-2a, -2b and -2c (Buonavoglia et al. 2001; Clegg et al. 2012; Nakamura et al. 2001), we cannot rule out the possibility that servals may be asymptomatic carriers of both FPLV and CPV-2b. Alternatively, servals may at times show mild transient intestinal infections with either virus that result in immune suppression, secondary bacterial enteritis and septicaemia. This could possibly explain the 2-week period between the initial gastrointestinal signs and the terminal septicaemia in this case (PV19). It is unclear what role the damaged bladder mucosa played in this case; it may have been the route of bacterial infection.

The presence of CPV-2a and CPV-2b in the samples collected from domestic dogs from South Africa 2 years before the serval case indicates the existence of these strains in the domestic dog population in the country, and this may also indicate a likely source of infection for the serval (PV19). Information on the contact or not between domestic dogs and this serval was not available. Further investigation of samples PV36, PV38 and PV40 may reveal whether or not these isolates represent unique variants of CPV-2a. The case of CPV-2c isolated from a domestic dog sample collected in Namibia is most likely an artefact of shortening the sequences to the shortest sequence in the data set, as this virus variant has not been reported in Africa or Australia (Decaro et al. 2005; Dogonyaro et al. 2013; Meers et al. 2007; Sykes 2013). Additionally, the results of the NCBI website based Basic Local Alignment Search Tool (BLAST) which compares an input sequence to the GenBank database of sequences, identified the full length sequence of this virus as CPV-2b.

Canine parvoviruses have spread widely across the globe since the 1970s because of contact with infected animals, inanimate fomites, flies or other reservoirs (Bagshaw et al. 2014; Barker & Parrish 2001). Over the last 15 years (2001–2016), however, very little information about the genetic diversity of parvoviruses and possible variants in South African domestic and non-domestic carnivores has been published. The epidemiology of these viruses is therefore largely unknown, including the transmission between domestic and non-domestic species and whether or not vaccination with live virus in either group affects the epidemiology. Natural recombination has been reported in porcine, mink and rodent parvoviruses (Shackelton & Hoelzer 2007). Similarly, recombination between FPLV and CPV-2, CPV-2a and CPV-2c, as well as vaccine and field strains has been reported but the exact mechanism is unknown (Mochizuki et al. 2008; Ohshima & Mochizuki 2009; Pérez et al. 2014). Therefore, recombination remains an important factor to consider when studying the evolution and genetic diversity of parvoviruses. Extensive surveys of parvoviral strains present in wild South African carnivores, as well as domestic dogs and cats, will help determine the strain diversity, geographic and host species distribution as well as possible sources of infection. How this recombination mechanism affects vaccine efficacy is largely unknown. As it has been previously shown that domestic cats, cheetah, tiger and serval (at present) are susceptible to both CPV and FPLV, the revision of currently accepted vaccination strategies, which primarily involves vaccinating felids against FPLV only, is required.
